# Dynamic Alterations in Functional Connectivity Density in Amyotrophic Lateral Sclerosis: A Resting-State Functional Magnetic Resonance Imaging Study

**DOI:** 10.3389/fnagi.2022.827500

**Published:** 2022-03-15

**Authors:** Jia-Yan Shi, Li-Min Cai, Jia-Hui Lin, Zhang-Yu Zou, Xiao-Hong Zhang, Hua-Jun Chen

**Affiliations:** ^1^Department of Radiology, Fujian Medical University Union Hospital, Fuzhou, China; ^2^Department of Neurology, Fujian Medical University Union Hospital, Fuzhou, China

**Keywords:** amyotrophic lateral sclerosis, dynamic, functional connectivity density, resting-state, functional magnetic resonance imaging

## Abstract

**Background and Aims:**

Current knowledge on the temporal dynamics of the brain functional organization in amyotrophic lateral sclerosis (ALS) is limited. This is the first study on alterations in the patterns of dynamic functional connection density (dFCD) involving ALS.

**Methods:**

We obtained resting-state functional magnetic resonance imaging (fMRI) data from 50 individuals diagnosed with ALS and 55 healthy controls (HCs). We calculated the functional connectivity (FC) between a given voxel and all other voxels within the entire brain and yield the functional connection density (FCD) value per voxel. dFCD was assessed by sliding window correlation method. In addition, the standard deviation (SD) of dFCD across the windows was computed voxel-wisely to measure dFCD variability. The difference in dFCD variability between the two groups was compared using a two-sample *t-*test following a voxel-wise manner. The receiver operating characteristic (ROC) curve was used to assess the between-group recognition performance of the dFCD variability index.

**Results:**

The dFCD variability was significantly reduced in the bilateral precentral and postcentral gyrus compared with the HC group, whereas a marked increase was observed in the left middle frontal gyrus of ALS patients. dFCD variability exhibited moderate potential (areas under ROC curve = 0.753–0.837, all *P* < 0.001) in distinguishing two groups.

**Conclusion:**

ALS patients exhibit aberrant dynamic property in brain functional architecture. The dFCD evaluation improves our understanding of the pathological mechanisms underlying ALS and may assist in its diagnosis.

## Introduction

Amyotrophic lateral sclerosis (ALS) is a major motor neuron disease in adult that involves progressive upper and lower motor neuron degeneration, thereby resulting in muscular weakness (Hardiman et al., 2017). Besides major motor symptoms, clinical and neuroimaging evidence confirm widespread extra-motor neurodegenerative involvement in ALS (Masrori and Van Damme, 2020). After the onset of symptoms, the mean duration of survival is usually 3 to 5 years (Lanznaster et al., 2018). So far, the therapeutic options for ALS are limited, and timely diagnosis is crucial for improving clinic symptoms and prolonging survival (Lanznaster et al., 2018). While the etiology and pathology of ALS are still unclear, it is imperative to develop new neuroimaging biomarkers for timely and accurate diagnosis of ALS.

Resting-state functional magnetic resonance imaging (fMRI) is a non-invasive neuroimaging technique that is based on measuring blood oxygen level dependent (BOLD) signals and has gained increased attention in neuroscience investigations (Smith, 2012). Resting-state fMRI can detect spontaneous neural activity in cortical and sub-cortical regions, when no specific task is performed (Lv et al., 2018). In fact, neuroimaging methods using resting-state fMRI allow the study of brain activity and improve our understanding of the pathophysiological mechanisms underlying ALS. For example, this method has shown that patients with ALS have reduced amplitude of low-frequency fluctuations (ALFFs), an index of regional neural activity within the left precentral gyrus as well as left middle occipital gyrus (Ma et al., 2020), and modified regional homogeneity (ReHo), an index of regional coherence of neural activity in the sensorimotor cortex (Bueno et al., 2019). Furthermore, resting-state fMRI can be used to study and assess variations in functional connectivity (FC) among distinct brain regions. The FC method has been used in ALS patients and has provided more information on ALS-related changes in brain functional architecture in the resting-state and may potentially be utilized as biomarker for ALS. For example, investigations based on resting-state fMRI have reported that patients with ALS exhibit disruption of FC within motor-related regions and in extra-motor areas, including the sensorimotor cortex, occipital cortex, temporal cortex, and insula (Chenji et al., 2016; Li et al., 2018).

The abovementioned studies on ALS are based on the major assumption that resting-state fMRI signals are stationary during scanning. However, dynamic changes in brain activity may occur over time (Calhoun et al., 2014). In fact, various complex cognitive and motor functions are associated with the brain function of dynamically integrating and coordinating information over time (Vidaurre et al., 2017). Resting-state fMRI-based dynamic FC analysis may be employed to reflect the dynamic properties of brain function as well as improve our understanding of brain development/maturation/aging (Chen et al., 2018) and cognitive-behavioral (Nomi et al., 2017) mechanisms. Furthermore, the utilization of dynamic FC analysis enables us to elucidate the mechanisms underlying several psychiatric and neurological disorders such as schizophrenia (Sendi et al., 2021a) and Alzheimer’s disease (Sendi et al., 2021b). Several studies have assessed FC changes in ALS using a dynamic point of view. In one recent resting-state fMRI study, individuals with ALS exhibited altered temporal traits of functional network connectivity, which is an informative variation of FC, between sensorimotor network and default mode/cognitive-control networks (Chen et al., 2021). Another investigation has found increased dynamic FC stability in the left sensorimotor cortex and right temporal pole, whereas decreased functional stability was observed in the right middle/inferior frontal gyrus in ALS patients (Wei et al., 2021). In addition, changes in dynamic FC property were correlated to ALS disease severity (Wei et al., 2021). These studies have demonstrated that dynamic FC analysis has the potential to uncover underlying mechanisms of ALS and facilitate in the development of new neuroimaging biomarker.

Functional connectivity density (FCD) analysis is an extension of the FC method that calculates the total FC strength of a given node with all other nodes within a whole-brain network (Lv et al., 2018). Nodes with higher FCD value suggest that these may be more important in functional integration (Tomasi and Volkow, 2010). Recently, the dynamic FCD (dFCD) approach, which combines FCD analysis and the sliding window correlation method, has also been employed to explore dynamic neural communication among distinct brain regions and thus has drawn increasing attention. For example, by calculating the standard deviation (SD) of FCD values across sliding windows, it is possible to quantify dFCD variability, which reflects abnormalities in brain dynamics in individuals diagnosed with psychiatric and neurological disorders, including generalized anxiety disorder (Chen et al., 2020) as well as epilepsy (Li et al., 2019). The current study aimed to investigate the altered dynamic properties of brain function in ALS by conducting the first assessment of dFCD variability to further provide the new insights into the mechanisms underlying ALS disease.

## Materials and Methods

### Participants

This study received approve from the Research Ethics Committee of Fujian Medical University Union Hospital. All subjects provided their written informed consent. A total of 50 patients who received a diagnosis of sporadic ALS and 55 healthy controls participated in this study. The El Escorial criteria (Brooks et al., 2000) were utilized in diagnosing ALS, with disease severity assessment by the revised ALS Functional Rating Scale (ALSFRS-R). Duration of ALS disease was also noted, with the rate of disease progression calculated using the following equation: (48-ALSFRS-R)/disease duration. The two study groups were matched in terms of age, gender, and educational attainment (Table 1). The following exclusion criteria were employed: (1) other neuropsychiatric disorders such as Alzheimer’s disease, epilepsy, Parkinson’s disease, or depression; (2) received psychotropic medications; (3) had co-morbidities such as respiratory failure or other chronic disorders such as heart failure and cancer; and (4) with contraindications of MRI examination.

**TABLE 1:** Subjects’ demographic and clinical information.

	Healthy controls (*n* = 55)	ALS patients (*n* = 50)	*P-*value
Age (years)	52.8 ± 6.9	52.3 ± 9.0	0.99
Gender (females/males)	19/36	19/31	0.71
Education (years)	7.8 ± 3.4	7.3 ± 3.9	0.40
Site of onset (bulbar/cervical/lumbosacral)	–	8/33/9	–
Diagnostic category (definite/probable/possible)	–	11/22/17	–
ALSFRS-R score	–	40.7 ± 5.3	–
Disease duration (months)	–	17.4 ± 16.1	–
Disease progression rate	–	0.64 ± 0.59	–

*ALS, amyotrophic lateral sclerosis; ALSFRS-R, revised ALS Functional Rating Scale. “–”, no data available.*

### Acquisition of Magnetic Resonance Imaging Data

Magnetic resonance imaging data acquisition was performed using a 3.0T scanner (Prisma, Siemens Medical Systems, Erlangen, Germany). We employed the multiband slice acquisition technique to capture resting-state functional images with the echo-planar imaging sequence, and the following parameters were used: multiband factor = 4, repetition time = 700 ms, acquisition matrix = 76 × 76, echo time = 30 ms, flip angle = 50°, slice thickness = 3 mm (no interslice gap), field of view = 228 mm × 228 mm, 600 volumes, and 48 axial slices. We asked the participants to have their eyes closed and to refrain from thinking about anything specific, and to remain still. For spatial normalization of functional images, we captured three-dimensional T1-weighted images (resolution = 1 mm^3^) using the magnetization prepared rapid gradient echo (MPRAGE) sequence, with the following parameters: repetition time = 1.61 s, echo time = 2.25 ms, acquisition matrix = 224 × 224, field of view = 224 × 224 mm, flip angle = 8°, slice thickness = 1.0 mm, and 176 slices.

### Functional Magnetic Resonance Imaging Data Preprocessing

Preprocessing of functional MRI data was performed using Statistical Parametric Mapping software (SPM^1^) and the Data Processing and Analysis of Brain Imaging toolbox (DPABI^2^). The first 30 volumes were discarded to allow signal equilibration and to give time for the participants to adapt to the scanning noise. We conducted realignment to correct head motion at various time points. No participants showed head movement >2.0 mm of translation or 2.0 degrees of rotation in the *x*, *y*, and *z* directions. Frame-wise displacement (FD), which shows volume-to-volume changes in head position, was also determined. The functional data consisting of the final sample were within the established motion thresholds of mean FD <0.25 mm. In addition, no significant difference (*P* = 0.12) in the mean FD value between the HC (0.07 ± 0.03 mm) and ALS (0.08 ± 0.04 mm) groups was observed. For normalization, individual structural images were initially co-registered using the mean functional image, with the transformed structural images segmented and then normalized into the Montreal Neurological Institute (MNI) space with a high-level non-linear warping algorithm, i.e., the Diffeomorphic Anatomical Registration Through Exponentiated Lie algebra (DARTEL) procedure (Ashburner, 2007). Then, we spatially normalized each functional volume to the MNI space using the deformation parameters that were estimated during the earlier step. After that, we processed the functional data using the linear trends as well as temporal band-pass filtering (0.01–0.08 Hz). Then, several nuisance covariates such as Friston 24-parameter, cerebrospinal fluid signal, and white matter signal were removed from the time series of all voxels by linear regression. As indicated in a previous study (Yan et al., 2012), global signal regression was not conducted because of concerns of increasing negative correlations (Weissenbacher et al., 2009) and potential distortions in group differences involving the intrinsic FC (Gotts et al., 2013).

### Analysis of Dynamic Functional Connection Density Variability

The analysis of dFCD was processed with the Temporal Dynamic Analysis (TDA) toolkit as implemented in DPABI. dFCD analysis was conducted using the sliding-window approach, with a window size of 90 TR (= 63 s) and a sliding step of 3 TR (= 2.1 s) (Yan et al., 2012). As controversies on the parameter settings of the sliding-window approach remain, the analyses with different window lengths (= 60 TR and 120 TR) as well as sliding steps (= 2 TR and 4 TR) were also conducted to assess reproducibility of our results. In every sliding window, the Pearson’s correlation coefficient (*r*) of each pair of brain voxels was calculated. This procedure was restricted to a gray matter mask, which was obtained using the automated anatomical labeling (AAL) atlas in the absence of the cerebellum (Li et al., 2019; Chen et al., 2020). We hence obtained a voxel-based whole-brain correlation for each voxel of every window. Then, we employed *r* = 0.25 as the correlation coefficient threshold to determine the connectivity between two voxels (Yan et al., 2012); this was performed to eliminate the weak correlations that were induced by noise. Then, in each window, the FCD, which is the weighted sum of positive correlations using each connection’s correlation coefficient *r* > 0.25, was calculated. Within the brain network, the FCD value of each node indicates its connectivity strength to all the other nodes and indicates its importance in functional integration.

To assess temporal variations in dFCD, the standard deviation (SD) of dFCD was calculated across the sliding windows following a voxel-wise manner. The SD maps were subsequently Z-standardized against their own mean and SD among all voxels within the gray matter mask. We then regarded these as the dFCD variability map of each subject. Lastly, we spatially smoothened individual dFCD variability maps using a 4-mm Gaussian kernel.

### Statistical Analysis

We used the non-parametric Mann–Whitney *U*-test in between-group comparisons of demographic variables, including mean FD index, age, and education level, with categorical variables (e.g., gender) compared with the chi-square test. Differences with *P* < 0.05 were deemed statistically significant.

For every group, the one-sample *t*-test was utilized on the dFCD variability maps to assess overall temporal variations. We used the two-sample *t-*test to detect differences in dFCD variability between the two groups using a voxel-wise approach. We set the statistical threshold to *P* < 0.05 corrected with the Gaussian random field (GRF) technique (voxel-level *P* < 0.001). Age, gender, educational level, as well as mean FD index were employed as covariates.

After conducting the two-sample *t-*test, we regarded areas with significant differences in dFCD variability as regions of interests (ROI). We then computed the mean dFCD variability of the ROIs. Then, by Sepearman correlation analysis, we assessed the correlation between dFCD variability and clinical parameters in the ALS patients, and those with a false discovery rate (FDR)-corrected *P-*value <0.05 deemed statistically significant. Furthermore, receiver operating characteristic (ROC) curve analysis was conducted to evaluate between-group discrimination performance of the mean dFCD variability index in different ROIs. In addition, the area under the ROC curve (AUC) was calculated using SPSS 20.0 (SPSS, Inc., Chicago, IL, United States).

## Results

**FIGURE 1:**
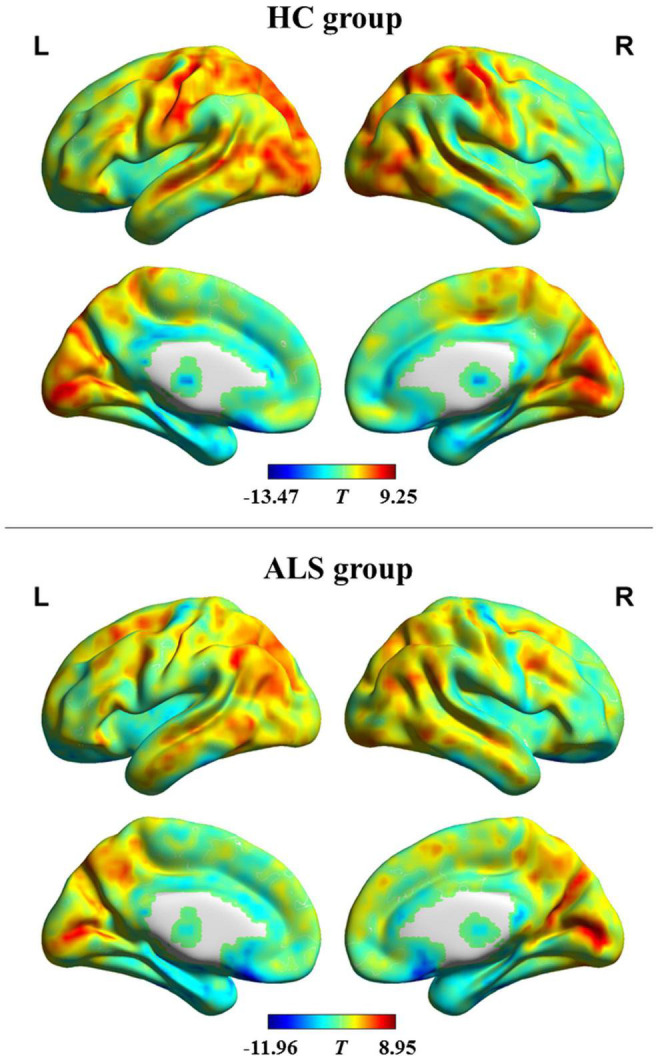
dFCD variability patterns within each group. The brain maps of the *T-*values indicate the results of one-sample *t-*testing of dFCD variability. High and low dFCD variability is represented by red and blue color, respectively. ALS, amyotrophic lateral sclerosis; HC, healthy control; L, left; R, right.

The pattern of dFCD variability of every group is shown in Figure 1. Using visual inspection, a set of brain areas showing relatively higher dFCD variability was bilaterally observed in the HC group, which mainly involve the parietal, motor-related, visual, somatosensory, and superior temporal cortices. However, several bilateral brain areas, including the medial and orbital frontal cortex, medial temporal cortex, cingulate cortex, insula, as well as subcortical regions, showed relatively lower dFCD variability.

**FIGURE 2:**
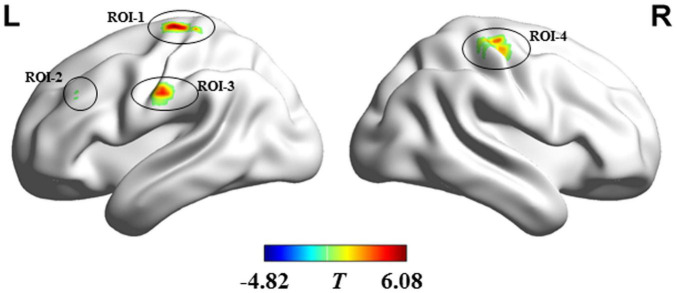
Regions showing between-group differences in dFCD variability. Regions in red and blue, respectively indicate decreased and increased dFCD variability in individuals with amyotrophic lateral sclerosis. ROI-1 and ROI-3 are located in the left precentral and postcentral gyri; ROI-2 is located in the left middle frontal gyrus; and ROI-4 is situated in the right precentral and postcentral gyri. L, left; R, right.

**FIGURE 3:**
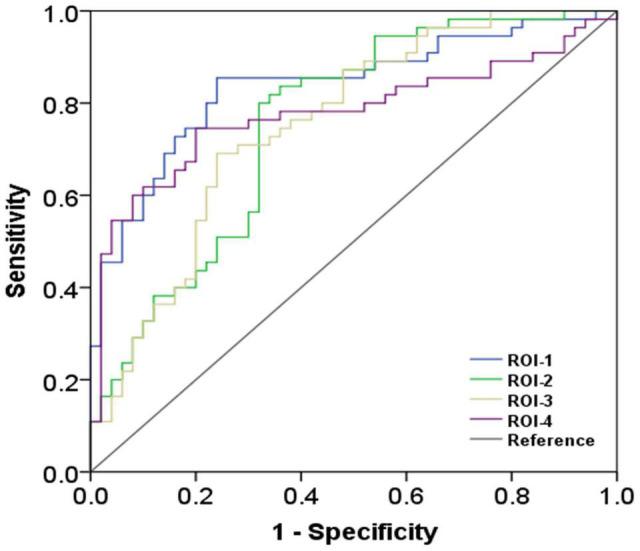
The findings of receiver operating characteristic curve analysis. ROI-1 and ROI-3 are located in the left precentral and postcentral gyri; ROI-2 is located in the left middle frontal gyrus; and ROI-4 is located in the right precentral and postcentral gyri (the details on the ROIs are in Figure 2 and Table 2).

Compared with the HC, ALS patients showed significantly reduced dFCD variability in the bilateral precentral and postcentral gyrus and showed significantly greater dFCD variability in the left middle frontal gyrus (Figure 2). In addition, analyses using different sliding-window parameter settings depicted highly similar patterns of between-group dFCD variability difference, indicating the robustness of our results (Supplementary Figure 1). No significant correlation between mean dFCD variability in ROIs and clinical parameters in ALS group was observed, after FDR correction. The results of ROC curve analysis are shown in Figure 3. The indices of mean dFCD variability in the ROIs (AUC for ROI-1–ROI-4 = 0.837, 0.753, 0.755, and 0.779, respectively; all *P* < 0.001) all depicted moderate potential in differentiating the two groups.

**TABLE 2:** Regions exhibiting between-group differences in dFCD variability.

Region	Voxels	Brodmann area	MNI coordinates			Peak *T-*value

			x	Y	z	
ROI-1: Left precentral and postcentral gyrus	37	4/6/3	−24	−21	69	6.08
ROI-2: Left middle frontal gyrus	22	9	−45	33	36	−4.82
ROI3: Left precentral and postcentral gyrus	22	4/3/6	−60	−15	33	4.60
ROI4: Right precentral and postcentral gyrus	34	4/3	36	−21	63	3.91

*ROI, region of interest.*

## Discussion

Our study investigated ALS-related alterations in brain dynamic property by assessing dFCD variability from a whole-brain perspective. The main findings were as follows: (i) The dFCD variability pattern observed in HC group is concordant to the findings of previous reports (Li et al., 2019; Chen et al., 2020); (ii) dFCD variability of ALS decreased in the bilateral precentral and postcentral gyrus, which represent brain regions implicated in sensorimotor function; (iii) patients with ALS exhibited increased dFCD variability in the left middle frontal gyrus, which is associated with cognitive and behavioral functions; and (iv) the indices of dFCD variability showed moderate discrimination power between the two groups, suggesting that it may potentially be used as a diagnostic biomarker for ALS.

Our finding of abnormal dFCD variability in ALS can be supported by the evidence from neurophysiological studies based on electroencephalography (EEG). By analyzing neural electrical signals from EEG, recent studies have shown altered FC patterns in ALS such as the increased synchronous EEG oscillations between the frontal and parietal regions (Nasseroleslami et al., 2019) and reorganization of inferior cortical network topology (Fraschini et al., 2016). Simultaneous resting-state EEG-fMRI investigations have revealed that the dynamic properties in FC that were detected by fMRI are closely correlated to EEG signals (Tagliazucchi et al., 2012). In these contexts, the abnormal dFCD variability in patients with ALS was not an unexpected finding.

The ALS patients had several regions with decreased dFCD variability and involved the sensorimotor cortex (i.e., the bilateral precentral and postcentral gyrus). Alterations in the primary motor cortex and premotor cortex are typical neuroimaging features of ALS (Menke et al., 2017). Previous studies have reported that the sensorimotor cortex is involved in ALS as evidenced by volume atrophy (Agosta et al., 2012), fiber degradation (Chio et al., 2014), and decreased N-acetyl aspartate/creatine ratio (NAA/Cr) or NAA levels (Buhour et al., 2017). Concordant to our findings, resting-state fMRI studies that employed the static connectivity analysis have revealed decreased FC within the sensorimotor network (Agosta et al., 2013; Chenji et al., 2016). Furthermore, in terms of dynamics, resting-state fMRI studies have observed reduced temporal variability in ALFF in the sensorimotor cortex (Ma et al., 2020). What’s more, an earlier resting-state fMRI study has indicated that ALS patients show an altered dynamic FC pattern between the sensorimotor network and the default mode network (Chen et al., 2021). Given that the sensorimotor cortex plays a key role in somatosensory and in performing and coordinating motor function, the altered dFCD variability in these regions may lead to somatesthesia and abnormal control of voluntary movements, which are often observed in ALS patients (Christidi et al., 2018).

Individuals with ALS show altered dFCD variability in the left middle frontal gyrus. In parallel, a positron emission tomography study in ALS observed decreased glucose metabolism in various brain regions, including the left middle frontal gyrus (Cistaro et al., 2014). Also, our finding is concordant to an earlier report that demonstrated decreased FC strength in the left prefrontal cortex (e.g., middle frontal gyrus) (Li et al., 2017). The left middle frontal gyrus has been implicated in cognitive functions, which include executive attention (Thomsen et al., 2004) and mnemonic responses (Leung et al., 2002). A growing body of research has found that the deficits in cognition (such as executive function and memory) (Beeldman et al., 2016) are involved in ALS. Therefore, we hypothesize that alterations in dFCD variability involving the left middle frontal gyrus are associated with impaired cognition in ALS.

The present study has several limitations. First, we conducted a cross-sectional design and did not assess the development of functional abnormalities during ALS progression. Second, the absence of analysis according to ALS subtypes prevented us from elucidating the effect of clinicopathological heterogeneity on the dynamic property of brain function. Third, the further comprehensive cognitive assessment could help us directly explore the relationship between altered dFCD variability and cognitive dysfunction in ALS. Forth, the ROC curve analysis was performed after that the ROIs were determined by between-group comparison; thereby, the concern about overfitting should be noted. Finally, the fixed time sliding window method applied in current study may not effectively capture the dynamics of functional collaboration among brain regions to some extent (Bansal et al., 2019). Future research can consider a more flexible dynamic window control approach [e.g., randomized window method (Zhu et al., 2021)].

Our results revealed abnormal dynamic patterns of brain functional architecture in ALS, which were reflected by the altered dFCD variability in the sensorimotor cortex and left middle frontal gyrus. dFCD evaluation could help us further understand the pathological mechanisms underlying ALS and may contribute to improved diagnosis.

## Data Availability Statement

The original contributions presented in the study are included in the article/Supplementary Material, further inquiries can be directed to the corresponding author.

## Ethics Statement

The studies involving human participants were reviewed and approved by Research Ethics Committee of Fujian Medical University Union Hospital. The patients/participants provided their written informed consent to participate in this study.

## Author Contributions

J-YS: data curation, formal analysis, investigation, and writing-review and editing. L-MC: data curation, formal analysis, validation, and roles/writing-original draft. J-HL: data curation, formal analysis, investigation, and roles/writing-original draft. Z-YZ: funding acquisition and investigation. X-HZ: formal analysis, investigation, and roles/writing-original draft. H-JC: conceptualization, formal analysis, supervision, project administration, funding acquisition, and writing-review and editing. All authors contributed to the article and approved the submitted version.

## Conflict of Interest

The authors declare that the research was conducted in the absence of any commercial or financial relationships that could be construed as a potential conflict of interest.

## Publisher’s Note

All claims expressed in this article are solely those of the authors and do not necessarily represent those of their affiliated organizations, or those of the publisher, the editors and the reviewers. Any product that may be evaluated in this article, or claim that may be made by its manufacturer, is not guaranteed or endorsed by the publisher.

## Footnotes

1
http://www.fil.ion.ucl.ac.uk/spm

2
http://rfmri.org/DPABI

## Supplementary Material

Supplementary Figure 1 The influence of various sliding-window parameter settings on dFCD variability analysis. The dynamic functional connectivity analysis was conducted using the sliding-window approach using distinct parameter settings: **(A)** 60 TR window size and 3 TR sliding step; **(B)** 120 TR window size and 3 TR sliding step; **(C)** 90 TR window size and 2 TR sliding step; and **(D)** 90 TR window size and 4 TR sliding step. The main findings of the between-group comparison could be reproduced using different window lengths and sliding steps, suggesting that various sliding-window parameter settings do not significantly alter the results.

## Abbreviations

ALS: amyotrophic lateral sclerosis
fMRI: functional magnetic resonance imaging
ALFF: amplitude of low-frequency fluctuations
FC: functional connectivity
FCD: functional connectivity density
SD: standard deviation
dFCD: dynamic functional connectivity density
ALSFRS-R: revised ALS functional rating
FD: frame-wise displacement
ROI: region of interest
ROC: receiver operating characteristic
AUC: the area under the ROC curve
EEG: electroencephalography.

## References

[B1] Agosta F. Canu E. Valsasina P. Riva N. Prelle A. Comi G. (2013). Divergent brain network connectivity in amyotrophic lateral sclerosis. *Neurobiol. Aging* 34 419–427. 10.1016/j.neurobiolaging.2012.04.015 22608240

[B2] Agosta F. Valsasina P. Riva N. Copetti M. Messina M. J. Prelle A. (2012). The cortical signature of amyotrophic lateral sclerosis. *PLoS One* 7:e42816. 10.1371/journal.pone.0042816 22880116 PMC3412820

[B3] Ashburner J. (2007). A fast diffeomorphic image registration algorithm. *Neuroimage* 38 95–113. 10.1016/j.neuroimage.2007.07.007 17761438

[B4] Bansal K. Garcia J. O. Tompson S. H. Verstynen T. Vettel J. M. Muldoon S. F. (2019). Cognitive chimera states in human brain networks. *Sci. Adv.* 5:eaau8535. 10.1126/sciadv.aau8535 30949576 PMC6447382

[B5] Beeldman E. Raaphorst J. Klein Twennaar M. de Visser M. Schmand B. A. de Haan R. J. (2016). The cognitive profile of ALS: a systematic review and meta-analysis update. *J. Neurol. Neurosurg. Psychiatry* 87 611–619. 10.1136/jnnp-2015-310734 26283685

[B6] Brooks B. R. Miller R. G. Swash M. Munsat T. L. World Federation of Neurology Research Group on Motor Neuron Diseases (2000). El Escorial revisited: revised criteria for the diagnosis of amyotrophic lateral sclerosis. *Amyotroph. Lateral Scler. Other Motor Neuron Disord.* 1 293–299. 10.1080/146608200300079536 11464847

[B7] Bueno A. P. A. Pinaya W. H. L. Rebello K. de Souza L. C. Hornberger M. Sato J. R. (2019). Regional Dynamics of the Resting Brain in Amyotrophic Lateral Sclerosis Using Fractional Amplitude of Low-Frequency Fluctuations and Regional Homogeneity Analyses. *Brain Connect.* 9 356–364. 10.1089/brain.2019.0663 30793923

[B8] Buhour M. S. Doidy F. Mondou A. Pelerin A. Carluer L. Eustache F. (2017). Voxel-based mapping of grey matter volume and glucose metabolism profiles in amyotrophic lateral sclerosis. *EJNMMI Res.* 7:21. 10.1186/s13550-017-0267-2 28266002 PMC5339262

[B9] Calhoun V. D. Miller R. Pearlson G. Adalı T. (2014). The chronnectome: time-varying connectivity networks as the next frontier in fMRI data discovery. *Neuron* 84 262–274. 10.1016/j.neuron.2014.10.015 25374354 PMC4372723

[B10] Chen H. J. Zou Z. Y. Zhang X. H. Shi J. Y. Huang N. X. Lin Y. J. (2021). Dynamic Changes in Functional Network Connectivity Involving Amyotrophic Lateral Sclerosis and Its Correlation With Disease Severity. *J. Magn. Reson. Imaging* 54 239–248. 10.1002/jmri.27521 33559360

[B11] Chen Y. Cui Q. Xie A. Pang Y. Sheng W. Tang Q. (2020). Abnormal dynamic functional connectivity density in patients with generalized anxiety disorder. *J. Affect. Disord.* 261 49–57. 10.1016/j.jad.2019.09.084 31600587

[B12] Chen Y. Liu Y. N. Zhou P. Zhang X. Wu Q. Zhao X. (2018). The Transitions Between Dynamic Micro-States Reveal Age-Related Functional Network Reorganization. *Front. Physiol.* 9:1852. 10.3389/fphys.2018.01852 30662409 PMC6328489

[B13] Chenji S. Jha S. Lee D. Brown M. Seres P. Mah D. (2016). Investigating Default Mode and Sensorimotor Network Connectivity in Amyotrophic Lateral Sclerosis. *PLoS One* 11:e0157443. 10.1371/journal.pone.0157443 27322194 PMC4913931

[B14] Chio A. Pagani M. Agosta F. Calvo A. Cistaro A. Filippi M. (2014). Neuroimaging in amyotrophic lateral sclerosis: insights into structural and functional changes. *Lancet Neurol.* 13 1228–1240. 25453462 10.1016/S1474-4422(14)70167-X

[B15] Christidi F. Karavasilis E. Rentzos M. Kelekis N. Evdokimidis I. Bede P. (2018). Clinical and Radiological Markers of Extra-Motor Deficits in Amyotrophic Lateral Sclerosis. *Front. Neurol.* 9:1005. 10.3389/fneur.2018.01005 30524366 PMC6262087

[B16] Cistaro A. Pagani M. Montuschi A. Calvo A. Moglia C. Canosa A. (2014). The metabolic signature of C9ORF72-related ALS: FDG PET comparison with nonmutated patients. *Eur. J. Nucl. Med. Mol. Imaging* 41 844–852. 10.1007/s00259-013-2667-5 24445987 PMC8957062

[B17] Fraschini M. Demuru M. Hillebrand A. Cuccu L. Porcu S. Di Stefano F. (2016). EEG functional network topology is associated with disability in patients with amyotrophic lateral sclerosis. *Sci. Rep.* 6:38653. 10.1038/srep38653 27924954 PMC5141491

[B18] Gotts S. J. Saad Z. S. Jo H. J. Wallace G. L. Cox R. W. Martin A. (2013). The perils of global signal regression for group comparisons: a case study of Autism Spectrum Disorders. *Front. Hum. Neurosci.* 7:356. 10.3389/fnhum.2013.00356 23874279 PMC3709423

[B19] Hardiman O. Al-Chalabi A. Chio A. Corr E. M. Logroscino G. Robberecht W. (2017). Amyotrophic lateral sclerosis. *Nat. Rev. Dis. Primers* 3:17071. 28980624 10.1038/nrdp.2017.71

[B20] Lanznaster D. de Assis D. R. Corcia P. Pradat P. F. Blasco H. (2018). Metabolomics Biomarkers: a Strategy Toward Therapeutics Improvement in ALS. *Front. Neurol.* 9:1126. 10.3389/fneur.2018.01126 30619076 PMC6305341

[B21] Leung H. C. Gore J. C. Goldman-Rakic P. S. (2002). Sustained mnemonic response in the human middle frontal gyrus during on-line storage of spatial memoranda. *J. Cogn. Neurosci.* 14 659–671. 10.1162/08989290260045882 12126506

[B22] Li F. Zhou F. Huang M. Gong H. Xu R. (2017). Frequency-Specific Abnormalities of Intrinsic Functional Connectivity Strength among Patients with Amyotrophic Lateral Sclerosis: a Resting-State fMRI Study. *Front. Aging Neurosci.* 9:351. 10.3389/fnagi.2017.00351 29163133 PMC5681965

[B23] Li R. Wang L. Chen H. Guo X. Liao W. Tang Y. L. (2019). Abnormal dynamics of functional connectivity density in children with benign epilepsy with centrotemporal spikes. *Brain Imaging Behav.* 13 985–994. 10.1007/s11682-018-9914-0 29956102

[B24] Li W. Zhang J. Zhou C. Hou W. Hu J. Feng H. (2018). Abnormal Functional Connectivity Density in Amyotrophic Lateral Sclerosis. *Front. Aging Neurosci.* 10:215. 10.3389/fnagi.2018.00215 30065647 PMC6056617

[B25] Lv H. Wang Z. Tong E. Williams L. M. Zaharchuk G. Zeineh M. (2018). Resting-State Functional MRI: everything That Nonexperts Have Always Wanted to Know. *AJNR Am. J. Neuroradiol.* 39 1390–1399. 10.3174/ajnr.A5527 29348136 PMC6051935

[B26] Ma X. Lu F. Chen H. Hu C. Wang J. Zhang S. (2020). Static and dynamic alterations in the amplitude of low-frequency fluctuation in patients with amyotrophic lateral sclerosis. *PeerJ* 8:e10052. 10.7717/peerj.10052 33194375 PMC7643554

[B27] Masrori P. Van Damme P. (2020). Amyotrophic lateral sclerosis: a clinical review. *Eur. J. Neurol.* 27 1918–1929. 10.1111/ene.14393 32526057 PMC7540334

[B28] Menke R. A. Agosta F. Grosskreutz J. Filippi M. Turner M. R. (2017). Neuroimaging Endpoints in Amyotrophic Lateral Sclerosis. *Neurotherapeutics* 14 11–23. 10.1007/s13311-016-0484-9 27752938 PMC5233627

[B29] Nasseroleslami B. Dukic S. Broderick M. Mohr K. Schuster C. Gavin B. (2019). Characteristic Increases in EEG Connectivity Correlate With Changes of Structural MRI in Amyotrophic Lateral Sclerosis. *Cereb. Cortex* 29 27–41. 10.1093/cercor/bhx301 29136131

[B30] Nomi J. S. Vij S. G. Dajani D. R. Steimke R. Damaraju E. Rachakonda S. (2017). Chronnectomic patterns and neural flexibility underlie executive function. *Neuroimage* 147 861–871. 10.1016/j.neuroimage.2016.10.026 27777174 PMC5303676

[B31] Sendi M. S. E. Zendehrouh E. Ellis C. A. Liang Z. Fu Z. Mathalon D. H. (2021a). Aberrant Dynamic Functional Connectivity of Default Mode Network in Schizophrenia and Links to Symptom Severity. *Front. Neural Circuits* 15:649417. 10.3389/fncir.2021.649417 33815070 PMC8013735

[B32] Sendi M. S. E. Zendehrouh E. Fu Z. Liu J. Du Y. Mormino E. (2021b). Disrupted Dynamic Functional Network Connectivity Among Cognitive Control Networks in the Progression of Alzheimer’s Disease. *Brain Connect.* [Epub Online ahead of print] 10.1089/brain.2020.0847 34102870 PMC10442683

[B33] Smith K. (2012). Brain imaging: fMRI 2.0. *Nature* 484 24–26. 10.1038/484024a 22481337

[B34] Tagliazucchi E. von Wegner F. Morzelewski A. Brodbeck V. Laufs H. (2012). Dynamic BOLD functional connectivity in humans and its electrophysiological correlates. *Front. Hum. Neurosci.* 6:339. 10.3389/fnhum.2012.00339 23293596 PMC3531919

[B35] Thomsen T. Specht K. Rimol L. M. Hammar A. Nyttingnes J. Ersland L. (2004). Brain localization of attentional control in different age groups by combining functional and structural MRI. *Neuroimage* 22 912–919. 10.1016/j.neuroimage.2004.02.015 15193622

[B36] Tomasi D. Volkow N. D. (2010). Functional connectivity density mapping. *Proc. Natl. Acad. Sci. U. S. A.* 107 9885–9890. 20457896 10.1073/pnas.1001414107 PMC2906909

[B37] Vidaurre D. Smith S. M. Woolrich M. W. (2017). Brain network dynamics are hierarchically organized in time. *Proc. Natl. Acad. Sci. U. S. A.* 114 12827–12832. 10.1073/pnas.1705120114 29087305 PMC5715736

[B38] Wei J. Lin J. H. Cai L. M. Shi J. Y. Zhang X. H. Zou Z. Y. (2021). Abnormal Stability of Dynamic Functional Architecture in Amyotrophic Lateral Sclerosis: a Preliminary Resting-State fMRI Study. *Front. Neurol.* 12:744688. 10.3389/fneur.2021.744688 34721270 PMC8548741

[B39] Weissenbacher A. Kasess C. Gerstl F. Lanzenberger R. Moser E. Windischberger C. (2009). Correlations and anticorrelations in resting-state functional connectivity MRI: a quantitative comparison of preprocessing strategies. *Neuroimage* 47 1408–1416. 10.1016/j.neuroimage.2009.05.005 19442749

[B40] Yan C. Yang Z. Colcombe S. Zuo X. Milham M. (2012). Concordance among indices of intrinsic brain function: insights from inter-individual variation and temporal dynamics. *Sci. Bull.* 62 1572–1584. 10.1016/j.scib.2017.09.015 36659475

[B41] Zhu Y. Li X. Qiao Y. Shang R. Shi G. Shang Y. (2021). Widespread plasticity of cognition-related brain networks in single-sided deafness revealed by randomized window-based dynamic functional connectivity. *Med. Image Anal.* 73:102163. 10.1016/j.media.2021.102163 34303170

