# Supplementary figures and images for: Dynamic Alterations in Functional Connectivity Density in Amyotrophic Lateral Sclerosis: A Resting-State Functional Magnetic Resonance Imaging Study
Source: Front Aging Neurosci. 2022 Mar 15;14:827500. doi: 10.3389/fnagi.2022.827500 (PMC8967369; doi:10.3389/fnagi.2022.827500)

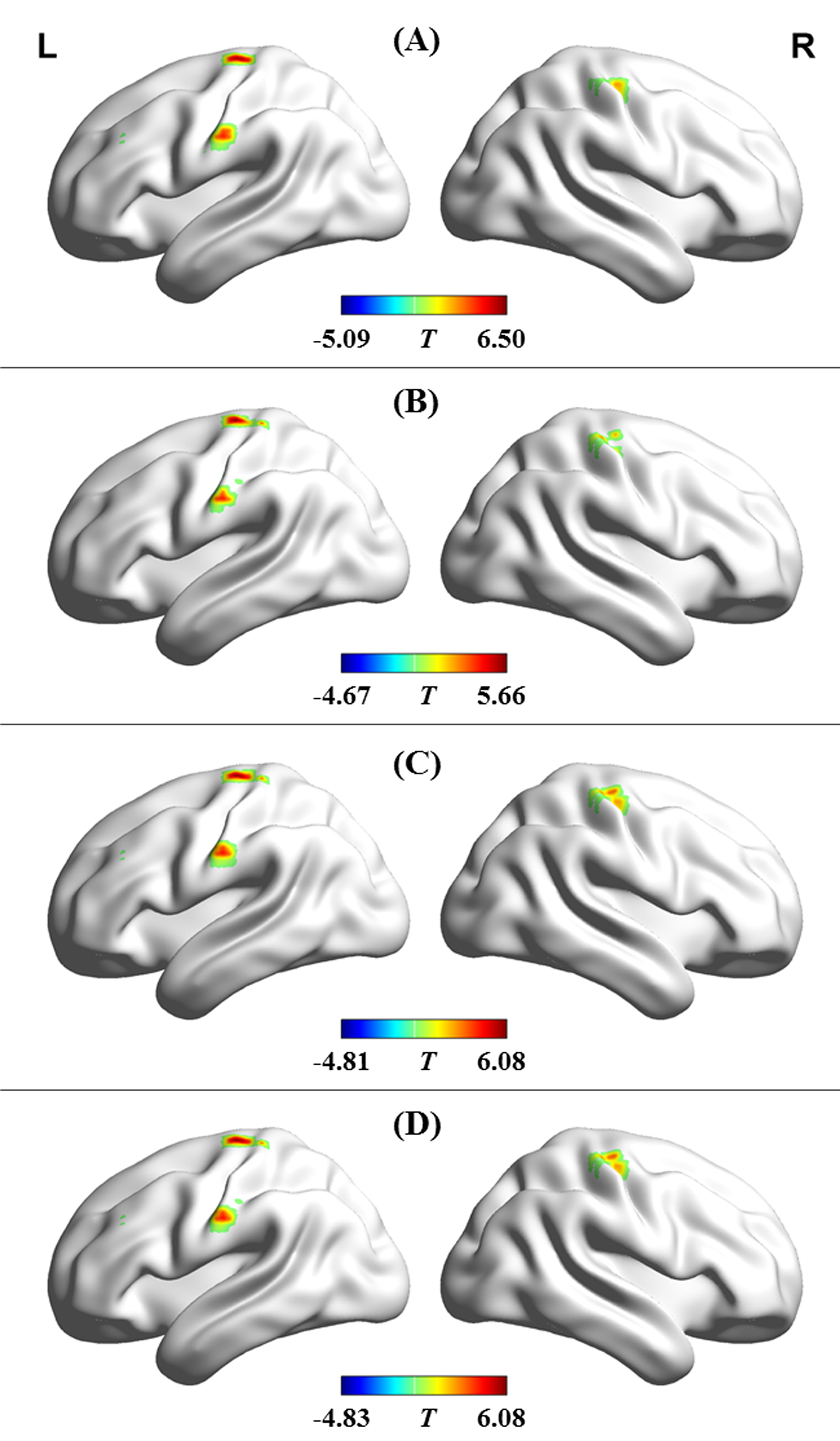

Supplement: Supplementary Figure 1 — The influence of various sliding-window parameter settings on dFCD variability analysis. The dynamic functional connectivity analysis was conducted using the sliding-window approach using distinct parameter settings: (A) 60 TR window size and 3 TR sliding step; (B) 120 TR window size and 3 TR sliding step; (C) 90 TR window size and 2 TR sliding step; and (D) 90 TR window size and 4 TR sliding step. The main findings of the between-group comparison could be reproduced using different window lengths and sliding steps, suggesting that various sliding-window parameter settings do not significantly alter the results. [file Image_1.TIF]
